# Adverse effects of antiretroviral therapy in pregnant women infected with HIV in Brazil from 2000 to 2015: a cohort study

**DOI:** 10.1186/s12879-018-3397-x

**Published:** 2018-09-27

**Authors:** Adriane M. Delicio, Giuliane J. Lajos, Eliana Amaral, Fabia Lopes, Fernanda Cavichiolli, Isabeli Myioshi, Helaine Milanez

**Affiliations:** 1 Department of Obstetrics and Gynecology, School of Medical Sciences, University of Campinas, Campinas, Brazil; 2 Department of Clinics, School of Medical Sciences, University of Campinas, Campinas, Brazil; 3 Referral Center for STIs and AIDS of Campinas, Campinas, Brazil

**Keywords:** HIV, Toxicity, Adverse effects, Antiretroviral therapy, Pregnancy

## Abstract

**Background:**

Antiretroviral therapy (ART) use in pregnancy presents unquestionable benefits in preventing mother-to-child transmission (MTCT) of HIV although it is associated with maternal adverse effects. The aim of this study was to evaluate the adverse effects of antiretroviral therapy in pregnant women infected with HIV.

**Methods:**

Cohort study of pregnant women infected with HIV followed at the CAISM/UNICAMP Obstetric Clinic from 2000 to 2015. The following maternal adverse effects were observed: anemia, thrombocytopenia, allergy, liver function test abnormalities, dyslipidemia and diabetes. Data collected from patients’ files was added to a specific database. Descriptive analysis was shown in terms of absolute (n) and relative (%) frequencies and mean, median and standard deviation calculations. Chi-square or Fisher exact test (*n* < 5) and relative risk (RR) with its respective *p* values were used for categorical variables and Student *t*-test (parametric data) or Mann-Whitney (non-parametric data) for the quantitative ones. A 95% confidence interval (CI) and a significant level of 0.05 were used. A multivariate Cox Logistic Regression was also done. Data analysis was conducted using SAS version 9.4.

**Results:**

Data from 793 pregnancies were included. MTCT rate was 2.3%, with 0.8% in the last 5 years. Maternal adverse effects were: dyslipidemia (82%), anemia (56%), liver function test abnormalities (54.5%), including hyperbilirubinemia (11.6%), fasting glycemia alteration (19.2%), thrombocytopenia (14.1%), and allergic reaction (2.7%). The majority of adverse effects deemed related to ART in this study were mild according to DAIDS scale. In the multivariate analysis, co-infections and starting ART during pregnancy were risk factors for maternal anemia, while CD4 count higher than 200 cells/mm^3^ was protective. Nevirapine, nelfinavir and atazanavir regimens increased the risk for liver function tests abnormalities. Lopinavir use during pregnancy increased the risk for fasting glycemia alteration.

**Conclusion:**

The evolution of the national guidelines of antiretroviral therapy for pregnant women improved adherence to the treatment and resulted in a significant reduction of MTCT. Despite the high frequency of maternal adverse effects, they are mostly of low severity. Newer ART medications with improved efficacy and significantly more favorable tolerability profiles should reduce the incidence of ART-related adverse effects.

## Background

The significant progress in antiretroviral treatment caused a striking decline in morbidity and mortality associated with *human immunodeficiency* virus (HIV) infection and a dramatic reduction in mother-to-child transmission (MTCT) [1–3]. Antiretroviral therapy for pregnant women is proven to be the most efficient intervention by reducing the rate of MTCT to lower than 2% and decreasing maternal and child mortality [4, 5].

The World Health Organization’s (WHO) Option B+ recommends the use of antiretroviral combined therapy (ART) to all pregnant and lactating women infected with HIV, independently from the CD4 count or the disease status. It also recommends maintaining its use after birth to control maternal disease and to prevent MTCT and sexual transmission of HIV [6]. A global treatment trend followed this recommendation not only in the developed countries but also in the ones with restricted economic resources. The Brazilian protocol follows the same guidelines to prevent MTCT of HIV [7].

Nevertheless, the increased use of potent and complex antiretroviral regimens during pregnancy can cause adverse effects in pregnant women and their newborns [8, 9]. The most frequent maternal adverse effects are hematologic, hepatic and dermatologic alterations, metabolic disturbances, pre-eclampsia, and viral resistance [10–14].

The Women’s Hospital at University of Campinas School of Medical Sciences (CAISM/UNICAMP) has been running a program for pregnant women infected with HIV since 1988. The objective of this study was to evaluate the ART maternal adverse effects in a large cohort of pregnant women infected with HIV followed at the Obstetric Clinic in this Brazilian public university hospital between 2000 and 2015.

## Subjects and methods

Observational analytic study based on the evaluation of a historic cohort in a population of pregnant women infected with HIV between 2000 and 2015 followed at the Obstetric Clinic at the University of Campinas School of Medical Sciences (CAISM/UNICAMP). CAISM is the reference hospital for high risk pregnancy in Campinas, the second biggest city in the State of São Paulo with 1.2 inhabitants and an Human Development Index (HDI) of 0.8. Mostly, CAISM serves pregnant women without health insurance and from low socioeconomic status. Follow-up of pregnant women infected with HIV at CAISM started in 1988. ART drugs started in 1994. The ART drug schemes varied in different periods: from 1994 to 1999 monotherapy with AZT; from 1999 to 2000 double AZT scheme with 3TC; from 2000 on, an association of AZT/3TC with a NNRTI (nevirapine) or a PI (nelfinavir). In 2006, nelfinavir was substituted for lopinavir/ritonavir (LPV/r). The AZT/3TC/LPV/r scheme used as a first line treatment for infected pregnant women since 2006 was substituted for TDF/3TC/EFV in 2015. By the end of 2017 efavirenz (EFV) was substituted for raltegravir (RAL) according to the new Brazilian guidelines for treatment. AZT was the drug of choice to compose the ART scheme during pregnancy until 2015 although tenofovir had been used as a substitute for AZT mainly in patients with anemia since 2010.

The women were selected from the clinical records in the medical files and from the epidemiologic Health Surveillance Agency data available in the clinic. A specific form was developed to collect all the information. The following variables were analyzed: pregnant women’s epidemiologic and clinical characteristics, antiretroviral treatment administrated, delivery characteristics, MTCT of HIV, adverse hematologic and hepatic effects, and glucose and lipids metabolic disorders. The time of pregnancy when antiretroviral treatment was introduced was described in terms of gestational weeks; women receiving ART in their last menstrual period were considered in treatment at conception. Peripartum viral load was measured at least 6 weeks prior to delivery. Substance use (cocaine/crack), alcohol use or smoking practice were considered previous or current in pregnancy. For the global analysis, the combination of antiretroviral drugs was classified as: monotherapy with nucleoside reverse transcriptase inhibitor (NRTI) zidovudine (AZT); double therapy with NRTI (AZT and lamivudine-3TC) or combined therapy (ART) defined as a combination of at least three drugs with no less than one protease inhibitor (PI) or one non-nucleoside reverse transcriptase inhibitor (NNRTI). Women using the PI darunavir (DRV), lopinavir (LPV) and atazanavir (ATV) also received booster of ritonavir (r). To evaluate the adverse effects, the main assessed outcomes were: laboratory abnormalities: anemia (hemoglobin < 11 g/dl), low platelets count (platelets < 150,000/ml), liver function test abnormalities (at least one: alanine aminotransferase-ALT > 34 U/L, aspartate aminotransferase-AST > 45 U/l, alkaline phosphatase-ALCPH > 104 U/l, gamma-glutamyl transferase*-*GGT > 42 U/l, bilirubin > 1,0 mg/dl); diabetes (glycemic curve with at least one abnormal value: fasting ≥92 mg/dl, after the first hour ≥180 mg/dl, after the second hour ≥152 mg/dl) and dyslipidemia (at least one abnormality: total cholesterol ≥200 mg/dl, triglyceride 150 ≥ mg/dl). Dyslipidemia criteria were used for adult population, not specified for pregnant women. Hemoglobin (Hb) level less than 11 g/dL was defined according to the Brazilian Health Ministry guidelines: mild (Hb 10–10,9 g/dL), moderate (Hb 8–9,9 g/dL) and severe (Hb ≤ 8 g/dL) [15].

The DAIDS Adverse Effect Scale (DAIDS AE) was used to quantify the severity of adverse effects. This grading scale provides an AE severity grading scale ranging from grades 1 to 5 with descriptions for each AE based on the following general guidelines: Grade 1 indicates a mild event, Grade 2 indicates a moderate event, Grade 3 indicates a severe event, Grade 4 indicates a potentially life-threatening event, Grade 5 indicates death [16].

All newborns exposed to HIV infection were followed by the Pediatric Immunodeficiency Service at the same University Hospital. The cases with no final HIV infection diagnosis due to loss of follow-up were contacted by phone or telegram. This study was approved by the Institution’s Ethics in Research Committee (Protocol #351/2006).

Descriptive analysis was shown in terms of absolute (n) and relative (%) frequencies, mean, median and standard deviation. Chi-square or Fisher exact test (*n* < 5) was used to analyze the association between the categorical variables. For the continuous variables, the Student *t*-test (parametric data) or Mann-Whitney (non-parametric data) were used. The specific effects of the different antiretroviral regimens were analyzed using relative risk (RR) with its respective *p* values. A multivariate Cox Logistic Regression was done. A 95% confidence interval (CI) and a significant level of 0.05 were used. Statistical analysis was performed using SAS version 9.4.

## Results

**Fig. 1:**
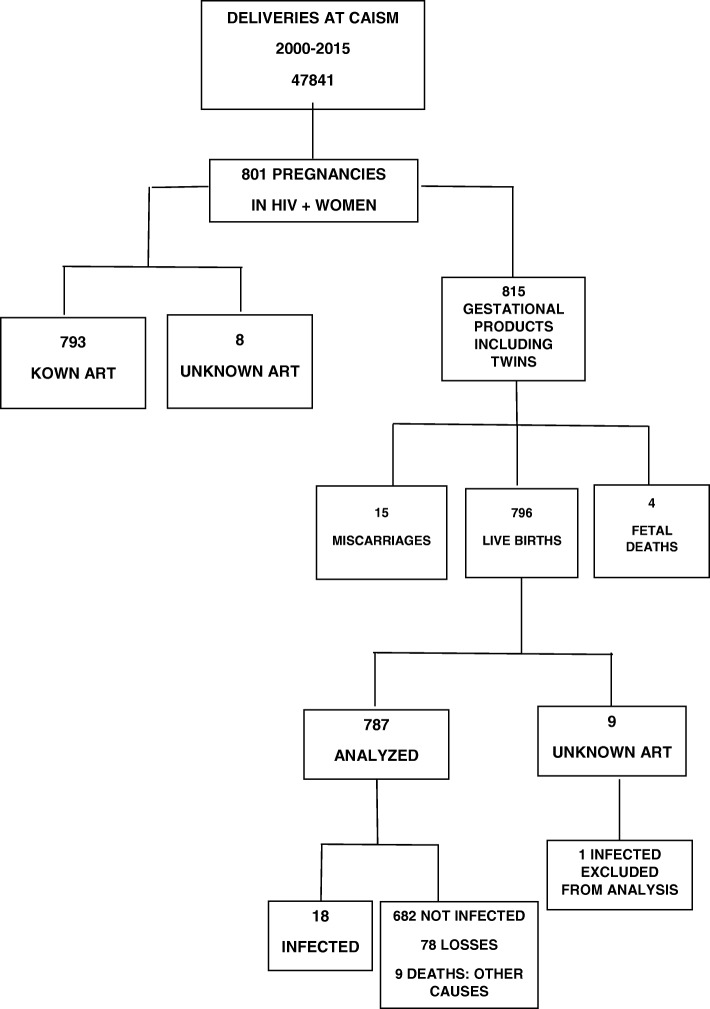
Flowchart of the study including pregnant women and newborns between 2000 and 2015. ART antiretroviral therapy

Between 2000 and 2015, there were 47,841 births at the university hospital. From these, 801 were pregnant women infected with HIV, with a 1.67% prevalence rate. Data referring to 14 multiple births were not replicated for the maternal analysis. Figure 1 shows all the eligible cases, the follow-up losses and the final number of 793 pregnancies and 787 children. The MTCT rate in this cohort was 2.3%. In the last 5 years of this study, of the 350 infected pregnant women, there were only three cases of HIV transmission, corresponding to a MTCT rate of 0.8%. For the few women who did deliver HIV positive infants three were in use of monotherapy with AZT, one was not taking any ART, one was in use of an unknown ART. The other ones were receiving three-drugs regimens.

Maternal adverse effects were: dyslipidemia (82%), anemia (56%), liver function test abnormalities (54.5%), including hyperbilirubinemia (11.6%), fasting glycemia alteration (19.2%), thrombocytopenia (14.1%), and allergic skin reaction (2.7%).

### Pregnant women, prenatal care and delivery characteristics

The mean age of the pregnant women was 28 years; 61.2% were white, and 60.9% completed elementary school. Pregnancy was planned in 25.7% of the cases, but only 27.6% were in use of antiretroviral therapy at conception. The exposure category of HIV was heterosexual in 93% of the women and 10 of them (2.5%) had acquired the infection through MTCT. Regarding the HIV diagnosis, 62% were aware of their serologic status and 40.7% had already used some antiretroviral regimen previously to this pregnancy. The most common ART regimens used during pregnancy are described in Table 1. Only 15 (1.9%) women did not use antiretroviral therapy while pregnant because the HIV diagnosis was made at admission for delivery. Eighty-one women (10.2%) got pregnant using Efavirenz (Table 1). The 81 patients in use of efavirenz in the first trimester had the drug changed to PI during prenatal care, except for one which kept it through the gestational period. Four pregnant women were using EFV at the end of the gestation, including three which started EFV in the second trimester. Five women used a combination of two NRTI with NVP and PI simultaneously (3 with LPV/r, 1 with ATV/r and 1 with NFV, included in their respective groups). AZT was changed to tenofovir (TDF) in 41 cases, to stavudine (d4T) in seven cases and to abacavir (ABC) in one case. The AZT combination with TDF cases (17 patients) were excluded from the specific analysis. The integrase strand inhibitor raltegravir (RAL) was added to the ART regimen in seven cases (four cases associated with LPV/r and three cases with DRV/r), mostly in the late gestational weeks with the goal of reaching undetectable viral load at delivery (Table 1).

**Table 1:** Baseline characteristics of the study population at CAISM/UNICAMP from 2000 to 2015

Characteristics	Median (%)
Age (years)	28 (13–46)
Parity	1 (0–9)
Schooling years (*n* = 769)	
< 8	60.9
8–11 yrs.	32.2
> 12	5.1
No schooling	1.8
Ethnicity: White	61.2
HIV diagnosis (*n* = 789)	
Prior to pregnancy	62.5
During pregnancy	37.5
In use of ART at conception (*n* = 787)	27.6
In use of Efavirenz at conception (*n* = 793)	10.2
Planned pregnancy (*n* = 382)	25.7
Heterosexual transmission (*n* = 408)	93.1
Baseline CD4 median (cells/mm^3^)	444 (3–1915)
Peripartum CD4 median (cells/mm^3^)	552 (5–2164)
Median time of ART use (days)	152.5 (4–292)
Median of detectable peripartum viral load (copies/ml)	815 (43–500.000)
Peripartum Viral Load < 50 (copies/ml) (*n* = 732)	58.9
CDC classification (*n* = 758)	
1	29.9
2	51.6
3	18.5
Substance abuse (*n* = 568)	14.3
Alcoholism (*n* = 528)	5.7
Smoking (*n* = 538)	14.1
ART adherence during prenatal care	84.3
Changed ART (*n* = 776)	19.2
Intravenous AZT (*n* = 775)	94.8
Antiretroviral regimens during pregnancy (*n* = 793)	
None	1.9
AZT monotherapy	2.9
Dual therapy	1.4
2 NRTI + 1 NNRTI	17.9
AZT + 3TC + NVP	16.8
TDF + 3TC + NVP	0.1
d4T + 3TC + NVP	0.5
AZT + 3TC + EFV	0.3
TDF + 3TC + EFV	0.3
2 NRTI + 1 PI	74.9
AZT + 3TC + LPV/r	48.5
TDF + 3TC + LPV/r	3.4
d4T + 3TC + LPV/r	0.1
TDF + AZT + 3TC + LPV/r	1.5
AZT + 3TC + ATV/r	1.8
TDF + 3TC + ATV/r	1.0
ABC + 3TC + ATV/r	0.1
TDF + AZT + 3TC + ATV/r	0.3
AZT + 3TC + NFV	16.8
d4T + 3TC + NFV	0.1
2 NRTI + Other PI*	1.3
2 NRTI + PI** + RAL	0.9
2 NRTI + NNRTI + PI	0.6
Total	100

*ART* antiretroviral therapy, *NRTI* nucleos(t)ide reverse transcriptase inhibitors, *AZT* zidovudine, *3TC* lamivudine, *TDF* tenofovir, d4T stavudine, *ABC* abacavir, *NNRTI* non-nucleoside reverse transcriptase inhibitor *NVP* nevirapine, *EFV* efavirenz, *PI* protease inhibitor, *NFV* nelfinavir, *LPV* lopinavir, *r* ritonavir, *ATV* atazanavir, *IDV* indinavir, *FPV* fosamprenavir, *SQV* saquinavir, *RAL* raltegravir, *CDC* Centers for Disease Control and Prevention

Other PI* IDV/r (6), FPV/r (2), SQV (2)

PI** DRV/r (3), LPV/r (4) included in LPV/r group

The median duration of antiretroviral therapy during pregnancy was 152.5 days (134.5 days for monotherapy with AZT, 231 days for NNRTI and 151 days for PI). The median baseline CD4 count was 444 cells/mm^3^ (varying from 3 to 1915) and the median perinatal CD4 count was 552 cells/ mm^3^. The median baseline viral load count was 1371 copies/ml. After ART 432 (58.9%) women reached undetectable viral load in the peripartum period (50.6% in the 2000–2009 period and 68.3% in the last 5 years of the study). Women with detectable peripartum viral load presented median of 815 copies/ml (Table 1).

The median number of prenatal appointments was 8 and the median gestational age at the beginning of prenatal care was 16 weeks. Two hundred and thirty pregnant women presented at least one obstetrical complication, mostly preterm labor, hypertension and intrauterine grow restriction. At least 667 women presented at least one infection during pregnancy: urinary infection, bacterial vaginosis, *Streptococcus* B colonization, hepatitis C, latent tuberculosis and syphilis (Table 2). Only four patients presented multiresistant virus in genotyping test (3.3%).

**Table 2:** Pregnancy outcomes at CAISM/UNICAMP from 2000 to 2015

Characteristics	Median (%)
Prenatal appointments	8 (0–18)
Gestational age at first appointment	16 (3–38)
Gestational age at birth	38 (22–42)
Obstetrical complications (*n* = 793)	29
Preterm labor	14.1
Hypertension	6.1
Intrauterine grow restriction	4.4
Pre-eclampsia	1.3
Co-infections during pregnancy (*n* = 787)	84.8
Urinary infection	34.7
Bacterial vaginosis	33.7
*Streptococcus* B colonization	33.4
Hepatitis C	7.7
Latent tuberculosis	5.5
Syphilis	5.2
Toxoplasmosis	2.3
Genital herpes simplex	2.1
Opportunistic disease during pregnancy (*n* = 785)	6.1
Active tuberculosis	1.7
Oral/esophageal candidiasis	1.7
Cerebral toxoplasmosis	0.9
Cytomegalovirosis	0.6
*Pneumocystis jirovecii*	0.4
Labor (*n* = 447)	42.1
Premature rupture of membranes (*n* = 773)	15.7
Preterm birth	21.7
Low birth weight	22.5
Total	100

Regarding to HIV infection during pregnancy, 51.3% of pregnant women were classified into the CDC stage 2 and 18.5% were classified as having Acquired Immunodeficiency Syndrome - AIDS (stage 3). Only 48 women (6.1%) presented opportunistic infections during pregnancy, mainly active tuberculosis and oral/esophageal candidiasis (Table 2).

Intravenous AZT was used in 94.8% of the cases. Regarding mode of delivery, in 92.8% of cases it was by cesarean, mostly due to the HIV infection, according to the hospital guidelines between 2005 to 2015. There were only four episiotomies in the vaginal births. Labor occurred in 42.1% of women, and 15.7% presented premature rupture of the membrane. The mean gestational age at birth was 38 weeks. Preterm birth occurred in 21.7% and 22.5% of the newborns presented low birth weight (Table 2).

All newborns received oral AZT. Twenty-four (3.1%) children received nevirapine associated with AZT in the first week of life to reinforce the neonatal prophylaxis. All women received cabergoline for lactation inhibition, but two children were breastfed due to maternal wish.

### Association between ART regimens and obstetrical characteristics

Women exposed to NVP presented lower infectious (*p* = 0.010, RR 0.60 CI 0.44–0.81) and obstetrical complications (*p* = 0.001, RR 0.6 CI 0.44–0.81), but higher risk of detectable viral load peripartum (*p* = 0.001, RR 1.70 CI 1.23–2.35), when compared to the ones using PI. In the same way, women exposed to NVP presented higher MTCT rate (RR 2.53 CI 1.37–4.65) compared to the ones exposed to PI. Regarding to protease inhibitors, women exposed to NFV presented lower risk for obstetrical complications (*p* < 0.0001, RR 0.42 CI 0.30–0.57), but higher peripartum detectable viral load (*p* = 0.014, RR 1.43 CI 1.06–1.95) compared to the ones using LPV/r. When evaluating the nucleosides (AZT) and nucleotides (TDF) analogues, women exposed to TDF presented increased risk for obstetrical complications (*p* = 0.033, RR 1.98 CI 1.04–3.77), when compared to the women exposed to AZT (data not shown).

### Maternal adverse effects

During pregnancy, 56% of the women presented anemia (27.5% in the first trimester and 40.8% in the third one), 54.5% showed increased hepatic enzymes, 14.1% had low platelet count (9.5% and 6.3% in the first and third trimester, respectively), 2.7% had allergic reaction, and 11.6% presented increased bilirubin in the third trimester. Dyslipidemia was observed in 82% of cases; of those, 67.9% had increased total cholesterol and 84.4% had elevated triglycerides. Increased fasting glycemia occurred in 19.2% of the women.

The majority of pregnant women developing an adverse effect deemed related to ART in this study had mild anemia (as classified by the DAIDS toxicity and Brazilian Ministry of Health guidelines [15, 16]), with most of cases of grade 2 anemia occurring in the 3rd trimester. Most of pregnant women presented mild hepatic alteration in both first and third trimesters. On the other hand, grades 2 and 3 dyslipidemia occurred more frequently in the third trimester. There was no difference in occurrence of adverse effects between two periods of study (2000–2009 and 2010–2015) (Table 3).

**Table 3:** Maternal ART-related adverse effects categorized using the DAIDS Toxicity Grading Scale according to periods of delivery

	2000–2009 (*n* = 452)	2010–2015 (*n* = 347)	p*
	%	%	
Hemoglobin First Trimester			
Grade 1	92.5	94.6	0.516
Grade 2	6.4	4.8	
Grade 3	1.1	0.6	
Hemoglobin Third Trimester			
Grade 1	83.6	84.9	0.916
Grade 2	15.5	14.6	
Grade 3	0.9	0.5	
AST First Trimester			
Grade 1	98.4	98.9	
Grade 2	0.7	0.7	1.000
Grade 3	0.7	0.4	
Grade 4	0.3	0.0	
AST Third Trimester			
Grade 1	99.4	100.0	
Grade 2	0.6	0.0	1.000
Grade 3	0.0	0.0	
Grade 4	0.0	0.0	
ALT First Trimester			
Grade 1	98.7	98.9	
Grade 2	0.7	0.7	1.000
Grade 3	0.3	0.4	
Grade 4	0.3	0.0	
ALT Third Trimester			
Grade 1	99.4	99.4	
Grade 2	0.6	0.6	1.000
Grade 3	0.0	0.0	
Grade 4	0.0	0.0	
Cholesterol First Trimester			
Grade 1	91.1	90.6	0.631
Grade 2	6.9	8.4	
Grade 3	2.0	1.0	
Cholesterol Third Trimester			
Grade 1	69.7	64.9	0.792
Grade 2	21.2	24.7	
Grade 3	9.1	10.3	
Triglyceride First Trimester			
Grade 1	95.5	94.2	0.820
Grade 2	4.5	5.3	
Grade 3	0.0	0.5	
Grade 4	0.0	0.0	
Triglyceride Third Trimester			
Grade 1	83.3	77.1	0.514
Grade 2	15.6	20.8	
Grade 3	1.0	2.1	
Grade 4	0.0	0.0	
Platelets First Trimester			
Grade 1	98.4	97.2	0.496
Grade 2	1.1	2.2	
Grade 3	0.0	0.3	
Grade 4	0.5	0.3	
Platelets Third Trimester			
Grade 1	99.5	98.8	0.480
Grade 2	0.5	0.5	
Grade 3	0.0	1.0	
Grade 4	0.0	0.0	
Bilirrubin First Trimester			
Grade 1	98.6	95.2	0.264
Grade 2	1.4	3.0	
Grade 3	0.0	1.8	
Grade 4	0.0	0.0	
Bilirrubin Third Trimester			
Grade 1	97.6	93.0	0.200
Grade 2	1.2	6.1	
Grade 3	1.2	0.9	
Grade 4	0.0	0.0	
Total	100	100	

*ALT* alanine aminotransferase, *AST* aspartate aminotransferase

During pregnancy, 19.2% of ART were changed as shown on Table 1. AZT was changed to tenofovir (TDF) in 41 cases, to stavudine in seven cases and to abacavir in one case, because of anemia. NVP was changed in 11 cases (9 for ART medication-related drug hypersensitivity/allergic reaction, with three of them Stevens-Johnsons Syndrome). The adverse effects were mild or moderate in most of cases. None was associated with maternal mortality during pregnancy or after birth and subsided after change. EFV was changed in 80 cases when the risk for fetal malformation associated with it was considered high. Later, it was proved safe. All cases of stavudine and/or didanosine use were substituted preventively, due to their high risk of severe adverse effects during pregnancy. After delivery, all women were referred to the infectious disease clinic for follow-up. Allergic reaction was associated with ART in 22 cases, 18 of which associated with nevirapine use. Most of the allergy cases were mild or moderate (grades 1 or 2) skin/allergic hypersensitivity reactions. In 9 cases, NVP was substituted for protease inhibitor (including three cases of grade 4 Stevens-Johnsons Syndrome according to DAIDS Scale) and the symptoms subsided.

Co-infection with hepatitis C virus (61 women) did not show a significant association with hepatic alteration (*p* = 0.063) or ART-related allergic reaction (*p* = 0.065). Gestational CD4 values higher than 250 cells/mm^3^ in the baseline (RR 0.91 CI 0.71–1.16) and in the last count (RR 0.94 CI 0.71–1.24) were not associated with a higher risk for hepatic changes. The pregnant women exposed to nevirapine with CD4 > = 250 cells/mm^3^ did not present a higher occurrence of hepatic alteration (*p* = 1.00) or ART medication-related allergic reaction (*p* = 1.00) (data not shown).

In the multivariate analysis, the occurrence of co-infections during gestation increased the risk of developing maternal anemia (RR 1.52 CI 1.04–2.21). Having a peripartum CD4 higher or equal to 200 cells/mm^3^ was associated with having a lower occurrence of ART-related anemia (RR 0.67 CI 0.51–0.90) (Table 5).

### Association between ART regimens and maternal adverse effects

Pregnant women exposed to NVP presented higher risk for elevated hepatic enzymes (*p* < 0.0001, RR 4.26 CI 2.67–6.81) and for allergic reaction (*p* = 0.002, RR 2.71 CI 1.69–4.37), but lower risk for dyslipidemia (*p* = 0.015, RR 0.56 CI 0.35–0.89) and for alteration in fasting glycemia (*p* = 0.032, RR 0.55 CI 0.31–0.97), when compared with the ones using PI (Table 4).

**Table 4:** Comparative analysis between pregnant women in use of NNRTI and PI

	NNRTI		PI		p*	RR	CI 95%
	n	%	n	%			
Co-infections during pregnacy					0.010		
Yes	111	78.2	515	86.7		0.60	0.44–0.81
No	31	21.8	79	13.3		1.00	
Obstetrical complications					0.001		
Yes	57	40.1	334	56.0		0.60	0.44–0.81
No	85	59.9	262	44.0		1.00	
ART adherence during pregnancy					0.387		
Yes	121	86.4	494	83.4		1.21	0.78–1.88
No	19	13.6	98	16.6		1.00	
ART changes during pregnancy					< 0.0001		
Yes	10	7.1	134	22.5		0.31	0.17–0.58
No	131	92.9	462	77.5		1	
Viral load after ART					0.001		
Undetectable	57	47.5	365	63.4		1	
Detectable	63	52.5	211	36.6		1.70	1.23–2.35
Anemia					0.664		
Yes	73	58.4	323	56.3		1.07	0.78–1.48
No	52	41.6	251	43.7		1	
Thrombocytopenia					0.479		
Yes	15	12.1	83	14.5		0.08	0.51–1.38
No	109	87.9	488	85.5		1	
Hepatic alteration					< 0.0001		
Yes	94	83.2	267	47.8		4.26	2.67–6.81
No	19	16.8	292	52.2		1	
Dyslipidemia					0.015		
Yes	52	72.2	406	83.9		0.56	0.35–0.89
No	20	27.8	78	16.1		1	
Allergic reaction					0.002		
Yes	10	7.5	11	1.9		2.71	1.69–4.37
No	123	92.5	578	98.1		1	
Fasting glycemia alteration					0.032		
Yes	12	11.9	112	21.1		0.55	0.31–0.97
No	89	88.1	418	78.9		1	
Total	142	100	597	100			

(#) Numbers are different due to the lack of information on some patients

* Chi-square

*ART* antiretroviral therapy, *NNRTI* non-nucleoside reverse transcriptase inhibitor, PI protease inhibitor

In the protease inhibitors evaluation, women exposed to NFV presented increased risk for hepatic enzymes elevation (*p* < 0.0001, RR 1.90 CI 1.38–2.62) and for allergic reaction (*p* < 0.0001, RR 4.07 CI 3.19–5.20), but a lower risk for fasting glycemia alteration (*p* = 0.015, RR 0.56 CI 0.34–0.92), when compared with the ones exposed to LPV/r. Pregnant women exposed to ATV/r presented higher risk for elevated bilirubin (*p* = 0.0002, RR 5.17 CI 1.98–13.51) when compared to the ones exposed to LPV/r (data not shown).

In the nucleosides (AZT) and nucleotides (TDF) analogues comparison, there was no difference in adverse effects occurrence between AZT and TDF regimens (data not shown).

In the evaluation of the adverse effects associated with the timing of ART introduction, the women who initiated treatment during pregnancy presented increased risk for anemia (*p* = 0.0002), for hepatic changes (*p* = 0.003) and for allergic reaction (*p* = 0.025) than the ones who were already in use at conception (data not shown).

In the multivariate analysis, starting ART during pregnancy (RR 1.35 CI 1.02–1.80) increased the risk for developing maternal anemia. The use of NVP (RR 2.04 CI 1.58–2.63), NFV (RR 1.50 CI 1.15–1.97) and ATV (RR 1.94, CI 1.22–3.08) increased the risk for hepatic alterations in pregnancy. The use of LPV (RR 2.08 CI 1.07–4.04) increased the risk for alteration in fasting glycemia during pregnancy (Table 5).

**Table 5:** Multivariate analysis of risk factors associated with maternal adverse effects

Risk Factor	Anemia				Relative Risk	Logistic Regression*	Hepatic alterations				Relative Risk	Logistic Regression*	Dyslipidemia				Relative risk	Fasting glycemia alterations				Relative risk	Logistic Regression*
	Yes		No				Yes		No				Yes		No			Yes		No			
	n	%	n	%	RR CI 95%	RR CI 95%	n	%	n	%	RR CI 95%	RR CI 95%	n	%	n	%	RR CI 95%	n	%	n	%	RR CI 95%	RR CI 95%
Co-infection during pregnancy																							
No	35	9.2	63	23.8	1	1																	
Yes	378	99.7	263	99.2	1.65(1.17–2.33)	1.52(1.04–2.21)																	
No information	1		2																				
Baseline CD4 (cells/mm3)																							
< 200	66	19.0	19	6.1	1		52	14.2	32	10.4	1		82	17.7	21	21.4	1	15	12.2	59	11.5	1	
> = 200	330	94.8	286	92.6	0.69(0.53–0.90)		315	85.8	276	89.6	0.86(0.64–1.16)		381	82.3	77	78.6	1.01(0.75–1.35)	108	87.8	453	88.5	0.95(0.55–1.63)	
No information	18		23				15		9				7		5			4		24			
Peripartum CD4 (cells/mm3)																							
< 200	59	16.6	13	4.1	1	1	43	11.7	29	9.4	1		65	14.0	13	13.1	1	12	9.8	48	9.4	1	
> = 200	337	94.9	291	92.4	0.66(0.50–0.86)	0.67(0.51–0.90)	323	88.3	281	90.6	0.9(0.65–1.23)		399	86.0	86	86.9	0.98(0.71–1.34)	111	90.2	464	90.6	0.97(0.53–1.75)	
No information	18		24				16		7				6		4			4		24			
CDC classification																							
1	97	30.6	117	55.5	1		107	28.8	96	30.5	1		141	30.2	28	27.5	1	39	31.0	154	29.4	1	
2	211	66.6	158	74.9	1.26(0.99–1.60)		191	51.3	160	50.8	1.03(0.82–1.31)		242	51.8	58	56.9	0.97(0.78–1.19)	63	50.0	274	52.3	0.93(0.62–1.38)	
3	95	30.0	41	19.4	1.54(1.16–2.05)		74	19.9	59	18.7	1.06(0.79–1.42)		84	18.0	16	15.7	1.01(0.77–1.32)	24	19.0	96	18.3	0.99(0.60–1.65)	
No information	11		12				10		2				3		1			1		12			
Peripartum viral load																							
Undetectable (< 50 copies/ml)	215	108.0	205	166.7	1								309	66.3	54	56.3	1	81	66.4	314	61.4	1	
Detectable (> = 50 copies/ml)	178	89.4	102	82.9	1.24(1.02–1.52)								157	33.7	42	43.8	0.93(0.77–1.12)	41	33.6	197	38.6	0.84(0.58–1.22)	
No information	21		21										4		7			5		25			
Start of ART use																							
Before pregnancy	58	16.3	80	32.3	1	1	58	15.4	77	24.3	1		97	20.7	21	20.4	1	37	29.6	95	18.0	1	1
During pregnancy	350	98.3	240	96.8	1.41(1.07–1.86)	1.35(1.02–1.80)	319	84.6	240	75.7	1.33(1–1.76)		371	79.3	82	79.6	1(0.80–1.25)	88	70.4	434	82.0	0.6(0.41–0.88)	0.67 (0.44–1.01)
No information	6		8				5						2		0			2		7			
NRTI																							
AZT	373	909.8	291	786.5	1																		
TDF	21	51.2	20	54.1	0.91(0.59–1.42)																		
No information	20		17																				
ART during pregnancy																							
NRTI + NRTI + NVP	72	21.1	49	17.6	1		92	26.0	17	5.6	2.04(1.58–2.63)	2.04(1.58–2.63)	52	11.5	19	19.8	1	10	8.3	87	17.5	1	1
NRTI + NRTI + NFV	77	22.5	50	17.9	1.02(0.74–1.41)		77	21.8	47	15.5	1.5(1.15–1.97)	1.5(1.15–1.97)	86	19.0	18	18.8	1.13(0.80–1.59)	15	12.4	99	19.9	1.28(0.57–2.84)	1.31(0.59–2.92)
NRTI + NRTI + LPV/r	228	66.7	183	65.6	0.93(0.72–1.22)		165	46.6	234	77.2	1	1	295	65.3	57	59.4	1.14(0.85–1.54)	91	75.2	292	58.8	2.31(1.20–4.43)	2.08(1.07–4.04)
NRTI + NRTI + ATV/r	11	3.2	14	5.0	0.74(0.39–1.40)		20	5.6	5	1.7	1.94(1.22–3.08)	1.94(1.22–3.08)	19	4.2	2	2.1	1.24(0.73–2.09)	5	4.1	19	3.8	2.02(0.69–5.91)	1.58(0.52–4.76)
No information	26		32				28		14				18		7			6		39			
Hepatitis C																							
No							345	90.6	299	94.3	1												
Yes							36	9.4	18	5.7	1.25(0.88–1.75)												
No information							1		0														

* Cox Logistic Regression

*ART* antiretroviral therapy, *NRTI* nucleos(t)ide reverse transcriptase inhibitors, *AZT* zidovudine, *TDF* tenofovir, *NVP* nevirapine, *NFV* nelfinavir, *LPV* lopinavir, *ATV* atazanavir, r ritonavir, *CDC* Centers for Disease Control and Prevention

## Discussion

In our study pregnant women infected with HIV presented significant rates of adverse effects, most of low severity. Anemia and hepatic alteration occurred in more than half of the cases and lower platelets count in almost 15%. Less than 3% presented ART-related allergic reaction.

The Obstetric Clinic in CAISM/UNICAMP is a reference service for HIV infected women in the Campinas region and serves a low-income population with less schooling. The great majority of the cases are highly complex including users of psychoactive substances (mainly crack). In this updated cohort of 15 years of medical care, the great majority of women were exposed to ART during pregnancy, delivered by C-section, received intravenous zidovudine during labor, did not breastfeed and their newborns received oral AZT for 6 weeks.

The antiretroviral drugs used in pregnant women during the study years varied according to the Brazilian Ministry of Health’s recommendations. Since 2000, an ART regimen with three combined drugs has been in use [7].

One unquestionable benefit of using ART during pregnancy is the viral load control which directly impacted MTCT. In our study, analyzing 793 pregnancies of women infected with HIV, the MTCT rate was 2.3%, close to the World Health Organization (WHO) Global Plan’s goal [17, 18]. Nevertheless, it is important to stress that among the 350 infected pregnant women in the cohort’s last 5 years, there were only three cases of MTCT, all from women with lower adherence to treatment and in abuse of substances. Previous studies from the same clinic showed MTCT rates of 2.9% in 2000 and 3.7% in 2009, the last one strongly related to no adherence to antiretroviral treatment during pregnancy [19, 20].

It is known that using complex and potent antiretroviral regimens during pregnancy can cause adverse effects for the mother and the newborn, including the organogenesis period [8, 9]. The physiologic changes in pregnancy affect the kinetics of absorption, distribution, biotransformation and elimination of medication, potentially altering pregnant woman’s susceptibility to the toxicity of different drugs [7]. These changes cause pharmacokinetic alterations with consequent reduction of exposure to most of the drugs during the gestational period. However, it is not always necessary to adjust their dosages until the end of pregnancy [21].

The most frequent and significant maternal adverse effects studied are hematologic, hepatic and dermatologic alterations, metabolic disturbances like diabetes mellitus and dyslipidemia, pre-eclampsia and viral resistance [8, 22].

Our study demonstrated a global anemia rate of more than 50% in pregnant women exposed to ART, which is in accordance with the international and national literature. Recent study showed high anemia occurrence in Brazilian pregnant women [23]. This adverse effect was not associated with any specific antiretroviral drug in our multivariate data analysis but was associated with the starting of ART during pregnancy, with the occurrence of infectious complications and with peripartum CD4 count lower than 200 cells/mm^3^.

It is known that anemia in pregnant women infected with HIV can be associated with other causes besides ART, such as advanced maternal disease, iron and folic acid deficiency, malnutrition, intestinal parasitosis, and increased intravascular volume (hemodilution); all significantly associated with increased risk for maternal mortality [24, 25].

The use of zidovudine is one of the causes of cytopenia in pregnant women infected with HIV. In general anemia occurs at the beginning of treatment, in the first 12 weeks of use, and it is frequently associated with low CD4 count and advanced disease [26–28], as demonstrated in our data.

Even when it is known that AZT is not the only cause of anemia in pregnancy, and that ART is linked to improved health, particularly in lower income countries, anemia is the most common adverse effect associated with AZT use in pregnancy, independently of the regimen [29–31]. A study with 1070 pregnant women in sub-Saharan Africa between 2005 and 2008 reported the same results [32]. Many studies have been demonstrating high rates of anemia independently from the treatment regimen, as the one in South Africa with 408 pregnant women and 64.2% rate of anemia [33]. Similarly, our multivariate analysis showed that low CD4 count is a risk factor associated with anemia.

Our data did not show a significant difference in the occurrence of anemia between pregnant women exposed to AZT or TDF regimens. We did not compare the use of monotherapy with AZT and combined ART because of the small number of cases using only AZT (23 cases). A clinical trial (PROMISE Trial) in six sub-Saharan African countries and India compared monotherapy with combined regimens (AZT/3TC/LPV/r or TDF/Emtricitabine/LPV/r) and demonstrated that maternal adverse effects were more frequent with combined regimens, although the difference was not significant [34].

A study with 117 pregnant women in 2011 demonstrated adverse hematologic and hepatic effects and gestational cholestasis particularly associated with nevirapine, zidovudine and nelfinavir use. The higher rates of discontinuation or substitution due to adverse effects or intolerance occurred with these same drugs, while only 1% of patients in use of lopinavir/ritonavir had the drugs changed [35]. Our data also evidenced an increased risk of elevated hepatic enzymes with the use of combined ART. In the multivariate analysis, the use of nevirapine was associated with increased risk for hepatic alteration during pregnancy, although NFV and ATV were also associated with these adverse effects.

In our cohort, nevirapine was the most utilized NNRTI. It was associated with increased hepatic alterations and hypersensitivity skin reactions, in agreement with recently published researches. Using NVP in pregnant women, especially with CD4 count higher or equal to 250 cells/mm^3^ is debatable, due to elevated risk for hepatic toxicity and skin allergic reaction already demonstrated [36, 37]. However, an elevated risk for hepatic alteration and allergic reaction was not demonstrated in pregnant women exposed to nevirapine with CD4 > = 250 cells/mm^3^ in our cohort. This drug is considered an alternative, seldom recommended for pregnant women in Brazil [7].

Data from a 2006 Brazilian study evaluating NVP adverse effects in 197 pregnant women reported a 5.5% toxicity rate, 4.5% of exanthema with only one case of Stevens-Johnson Syndrome, and 1.5% of hepatotoxicity including one severe case [38]. That study also described hepatitis C virus as the only risk factor for NVP toxicity, which was also demonstrated in recent studies [39, 40]. In our cohort, pregnant women co-infected with hepatitis C virus did not present higher hepatotoxicity due to ART use.

In our study, despite the low rates of maternal skin allergic reaction associated with NVP, there were three cases of Stevens-Johnson Syndrome with total recovery.

A clinical trial in Uganda compared LPV/r and efavirenz (EFV) use in 389 women infected with HIV during pregnancy and puerperium and found no difference in the adverse effects such as anemia and neutropenia related to the two drugs. On the other hand, the pregnant women exposed to EFV presented higher viral suppression at delivery compared to the ones in use of LPV/r, although this difference was extinguished one year after birth [41]. In our study only four pregnant women were using EFV by pregnancy’s conclusion and only one of them used it continuously from the beginning to the end. Although for years EFV has been largely used in adults’ antiretroviral regimens due mainly to its highly potent effects and its simpler posology, only recently and after its security was demonstrate, was this drug suggested as the preferable regimen for pregnant women [6, 42]. For this reason, in 2015 the Brazilian Health Ministry recommended EFV as the chosen drug for initial treatment [43]. Nevertheless, recent studies discussed its significant collateral effects, in special the neuropsychiatric one and the increased risk for suicide [44, 45], besides its lower potentiality when compared to the promising integrase strand inhibitors [46]. Because of this, the most recent Brazilian recommendation is to use raltegravir as first choice to complete ART regimen in pregnancy [7].

Until 2015 the main recommended drug for pregnant women in Brazil was lopinavir/ritonavir (LPV/r). Atazanavir/ritonavir (ATV/r), which was highly recommended in international guidelines, was restricted in Brazil to first line PI intolerance cases and was associated with higher risk for elevated bilirubin, without clinical implications to the newborn. Bilirrubin elevation is a known adverse effect of atazanavir during pregnancy or anytime of use. It is known that protease inhibitors can cause lipid metabolic changes, metabolic syndrome, lipodystrophy, changes in the carbohydrate metabolism and increased cardiovascular risk in every HIV infected patient [47–49]. Some studies had demonstrated that during pregnancy women in use of PI also presented higher rates of dyslipidemia, metabolic syndrome, lipodystrophy, diabetes and pre-eclampsia [50–52], as some of our data also demonstrated.

When evaluating the different ART classes, PI use was associated with elevated risk for dyslipidemia and fasting glycemia alterations when compared to NVP use. In the multivariate analysis, LPV use was associated with elevated risk for alteration in fasting glycemia, demonstrating the need for its early evaluation and diagnosis in the pregnant women exposed to PI, and the need to revise the criteria for indicating PI to women with known risk factors for diabetes, such as obesity.

On carbohydrates metabolism, a study showed that some antiretroviral drugs increased the TNFα, IL-6 e IL-1β pro-inflammatory *cytokine*s involved in changes of adipocytes function and in the decrease of adiponectin, a positive modulator for insulin sensitivity [53]. A multi-center randomized clinical trial with 1407 women observed a general rate of gestational diabetes in 2.1%, higher with PI use (4.6%) [54].

Regarding birth outcomes, the authors used the same pregnant women’s cohort and their newborns to analyze neonatal adverse effects such as prematurity, low birth weight, small for gestational age week, neonatal anemia and hepatic alterations. The results on birth outcomes are described in another article which has just been published [55].

The high Caesarian section rate in this cohort occurred because from 2005 to 2015 most of pregnant women infected with HIV delivered by C-section following changes in the service’s protocol after the European Collaborative Group published their study [56]. On the other hand, prematurity was not considered iatrogenic and was not associated with C-section as shown in the recently published article by the same authors [55].

Our study focus was on the evaluation of adverse effects to which we produced consistent data on a clinical cohort in use of ART following the Brazilian Health Ministry’s recommendations, using guidelines very similar to the international ones. Data collection based on information registered in the women’s and children’s clinical records might have contributed to a possible register bias. Another limitation was having no HIV negative controls. The authors did not compare adverse effects in HIV negative control women because some tests to evaluate toxicity (hepatic alterations and dyslipidemia) are done only when the pregnant woman is in use of specific medication such as ART.

Our data showed more than 700 evaluations of pregnant women infected with HIV followed in a specialized clinic showing that the ART choices recommended by the Brazilian Health Ministry were adequate, leading to low gravity adverse effects and reaching excellent rates of MTCT.

## Conclusions

Despite the growing evidence on the unquestionable benefits of antiretroviral treatment during pregnancy, the clinical monitoring on drug safety and efficacy is of extreme importance to optimize the treatment recommendations.

The evolution of the national guidelines of antiretroviral therapy for pregnant women improved adherence to the treatment and resulted in a significant reduction of mother-to-child transmission of HIV throughout the years. Despite the high frequency of maternal adverse effects, they are mostly of low severity. Parallelally, recommending newer ART medications with improved efficacy and significantly more favorable tolerability profiles (introduction of tenofovir/lamivudine plus raltegravir for first line) should reduce the incidence of maternal ART-related adverse effects, which will reduce maternal as well as infant morbidity and potentially mortality.

## Abbreviations

3TC: Lamivudine
ABC: Abacavir
AIDS: Acquired immunodeficiency syndrome
ALCPH: Alkaline phosphatase
ALT: Alanine aminotransferase
ART: Antiretroviral therapy
AST: Aspartate aminotransferase
ATV: Atazanavir
AZT: Zidovudine
CAISM/UNICAMP: Women’s Hospital at University of Campinas School of Medical Sciences
CI: Confidence interval
d4T: Stavudine
DRV: Darunavir
EFV: Efavirenz
FPV: Fosamprenavir
GGT: Gamma-glutamyl transferase
HIV: Human immunodeficiency virus
IDV: Indinavir
LPV: Lopinavir
MTCT: Mother-to-child transmission
NFV: Nelfinavir
NNRTI: Non-nucleoside reverse transcriptase inhibitor
NRTI: Nucleoside reverse transcriptase inhibitors
NVP: Nevirapine
PI: Protease inhibitor
r: Ritonavir
RAL: Raltegravir
RR: Relative risk
SQV: Saquinavir
TDF: Tenofovir
WHO: World Health Organization
